# Spatial Assessment of Land Suitability Potential for Agriculture in Nigeria

**DOI:** 10.3390/foods13040568

**Published:** 2024-02-14

**Authors:** Jeffrey Chiwuikem Chiaka, Lin Zhen, Yu Xiao, Yunfeng Hu, Xin Wen, Fabien Muhirwa

**Affiliations:** 1 Institute of Geographic Sciences and Natural Resources Research, Chinese Academy of Sciences, Beijing 100101, China; cjchiwuikem2019@igsnrr.ac.cn (J.C.C.); xiaoy@igsnrr.ac.cn (Y.X.); huyf@ireis.ac.cn (Y.H.); xinwen4@cunet.carleton.ca (X.W.); fmuhirwa2019@igsnrr.ac.cn (F.M.); 2 State Key Joint Laboratory of Environmental Simulation and Pollution Control, School of Environment, Beijing Normal University, Beijing 100875, China; 3 Anambra-Imo River Basin Development Authority, Agbala 460109, Imo State, Nigeria; 4 School of Resource and Environment, University of Chinese Academy of Sciences, Beijing 100049, China; 5 Department of Biology and Institute of Environmental and Interdisciplinary Science, Carleton University, Ottawa, ON K1S 5B6, Canada; 6 Xinjiang Institute of Ecology and Geography, Chinese Academy of Sciences, Beijing 100101, China

**Keywords:** land cover, environmental factors, cropland suitability, analytical hierarchal process, Nigeria

## Abstract

From land cover analysis, cropland expansion was a major driving factor for land use land cover changes in Nigeria from 2000 to 2020. This further highlights the food production needs in the country. While this land use change indicates a significant alteration in land cover, it was exigent to assess land suitability using a Multi-Criteria Decision Analysis (MCDA) combined with geospatial techniques to identify areas with agricultural suitability potential and to analyze cropland suitability. The results showed that the country had 8% of very high suitability, high suitability (25%), moderate suitability (29%), and marginal suitability (25%) croplands. However, low suitability accounts for 14% of the entire cropland. The spatial distribution of cropland suitability shows that most areas in the South East, South South, and South West, respectively, have the most suitable cropland as they meet the biophysical conditions for crop production, followed by the North Central regions, while most places in the North (North East and North West) have a higher share of moderate to low suitability. This study highlights the potential of the country to target localized self-sufficiency. Therefore, this study recommends using the cropland suitability map to launch food security programs across the six geopolitical zones to maximize their inherent environmental potentials to alleviate the country’s food production needs.

## 1. Introduction

The “Malthusian theory of population” postulates that population will exceed food production for its sustenance, eventually leading to food shortages, etc., [1]. By the turn of the 1950s, the world’s population had substantially increased, and the “Green Revolution” ushered in a period of improved global food security and reduced malnutrition [2]. However, in Africa, despite the progress made in food security between 2000 and 2010, the level of hunger has increased, particularly between 2019 and 2022 [3]. This means the number of people facing malnutrition and food insecurity has increased, and the case in Africa is considered severe [4]. Moreover, 75% of the world’s current population lives where most of the world’s existing poverty are recorded [5]. In addition, the United Nations’ statistics highlight that an estimated 75 million people were added to the people living in extreme poverty in 2022 [6]. Consequently, households unable to afford the food available in the market will contribute to exacerbate the problem of malnutrition [7]. Therefore, to ensure food availability through local production, the intensification of agriculture is inevitable to meet the growing demand for food and livelihood [8,9]. For brevity, 80% of the rural population in East Africa depends on agriculture for their livelihood, similar to most populated countries, such as in the Northern parts of China [10,11,12]. Furthermore, countries within the European Union use an estimated 67% of the total agricultural area for livestock production [13], and on a global perspective, 35% of land is used for meat production [14].

Based on the above, to find a solution to food supply challenges, countries rely on food imports as a food security strategy to support local food production [15]. However, this option is also dependent on economic purchasing power, and this measure may not be sustainable due to various factors such as population growth [16] compared to advocating for self-sufficiency [17]. For instance, Nigeria is currently the seventh largest population in the world and is projected to become the third largest by 2050 [18]. The country has significant food import expenditure [7], with rice importation increasing from US $259 million in 1999 to US $756 million in 2002 [19], and $2.41 billion was spent on rice importation between 2012 and 2015 [20]. Considering the Gini coefficient, which is a measure of income inequality, Nigeria has a Gini coefficient of 35 [21]. This means that access to food in Nigeria is low, as fewer people than the majority of the population can afford it. Hence, promoting local self-sufficiency from imported staple foods will help to improve household access to food.

Subsequently, previous studies associated the challenge of food production in Nigeria to inadequate knowledge and assessment of the suitability of lands for agricultural production [22,23], even though Nigeria, Ethiopia, and Tanzania were listed among 44 countries assessed as having the potential to increase food production in sub-Saharan Africa [24]. The emerging literature has shown that land suitability is an important factor in maximizing food production, and the land suitability assessment, especially for cropland at the national level in Nigeria, remains rather vague. Consequently, traditional methods of assessing land suitability include field transects, surveys, geophysical investigations, and questionnaires; these methods are often cumbersome and time-consuming. However, the use of Geographic Information System (GIS) and remote sensing data is on the rise due to their cost-effectiveness and fine spatiotemporal coverage. Hence, geoinformatics has given rise to precision agriculture in response to the nuanced geographic factors that influence crop growth conditions [25].

Various countries such as the Central Anatolia region of Turkey, Iran, Ethiopia, and Zimbabwe have attempted to assess their land suitability for agricultural production [26,27,28,29] by using primary and secondary data techniques such as remote sensing and in situ investigations as the use of a single factor has been criticized [25]. However, assessing land suitability potential for agriculture in Nigeria is an emerging frontier, employing GIS techniques and environmental factors as analytical proxies such as soil morphology, slope, geology, and land cover [23,30,31]. Consequently, land suitability studies have been conducted in Nigeria [25,32]. However, these studies have focused primarily on the assessment of land suitability for food production at the state level. Hence, there is a need for a national spatial assessment using fine-scales data resolution to evaluate the agricultural potential in Nigeria, specifically based on existing cropland. This research is significant because the knowledge about land suitability for food production contributes to addressing the issue of low food productivity, which contributes to malnutrition concerns for Nigeria [33]. Even a recent study by Chiaka et al., [34], indicates that smallholder farmers are not meeting food production expectations. Moreover, this study is imperative as studies in Africa and Asia have shown that an increase in food production has led to a reduction in poverty [35]. Furthermore, the COVID-19 pandemic has highlighted the vulnerability of food security in most developed countries due to demand [36], much more so developing countries.

In this research, the suitability of the existing cropland for food production in Nigeria is assessed based on selected spatial environmental, pedological, and climatic variables. The study applied a Multi-Criteria Decision Analysis (MCDA), combined with geospatial techniques, to analyze remotely sensed variables to classify cropland suitability areas based on agriculturally suitable characteristics at the national level, compared to previous studies that only considered the land suitability at the state level. Furthermore, the study aims to contribute by highlighting the percentage of existing cropland suitability across the six geopolitical zones in Nigeria. This information is intended to support food security programs and aid in decision-making processes related to agricultural planning and development.

## 2. Material and Methods

### 2.1. Overview of the Study Area

**Figure 1:**
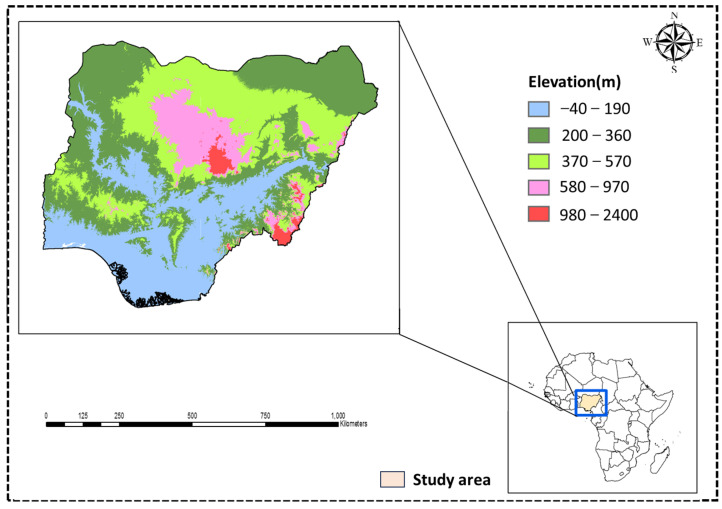
Map of digital elevation model and the study area location in Africa.

As one of the agrarian countries located in West Africa (Figure 1), agriculture contributes about 32% of non-oil earnings to Nigeria’s gross domestic product (GDP) despite the low irrigation of cultivated area [37]. In addition, the agricultural sector employs about two-thirds of Nigeria’s labor force [9] and serves as a foreign exchange earner for Nigeria. The discovery of crude oil contributed to a decline in agricultural produce export and turned the country into a net importer of food [38].

Nigeria has a tropical climate, with a rainy season mostly from April to October and a dry season from November to March. The average annual rainfall variation is above 2000 mm in the South and less than 500 mm in the North [39], and the precipitation distribution pattern decreases from the South to the North. The climate allows for the cultivation of various crops, with the cropping pattern such as rice, millet, cowpea, guinea corn, maize, and yams being cultivated more in the Northern area, while roots and tubers such as cassava, yam, and cocoyam, in addition to plantain, are grown in Southern areas. Additionally, oil palm, maize, and rice are also prominent crops cultivated in the Southern regions.

The Food and Agriculture Organization (FAO) estimates that Nigeria has about 70.8 million hectares of arable land [40]. As of 2020, the cropland area in the country showed that the Northern area has a total of 37,382,786 million hectares, and the Southern part encompasses 7,966,500 million hectares [41]. From Statista data [42], the percentage distribution of crops cultivated across the country in 2019 was as follows: rice (14.1%), maize (49%), millet (19.9%), guinea corn (29.6%), cowpea (20.9%), cassava (47%), yam (25.8%), and cocoyam (7.7%). Cash crops grown include cocoa, groundnuts, oil palm, and rubber [43].

### 2.2. Data Sources

This research was based on spatial datasets that include land use land cover, soil organic carbon (SOC), precipitation, land surface temperature (LST), elevation, slope, and surface soil moisture to classify cropland suitability areas (Table 1). The selection of these variables is similar to published land suitability studies [23,25,44], with an addition of the SOC parameter. All data were processed using ArcGIS 10.6 and Google Earth Engine. Also, the datasets were resampled to 30 m spatial resolution.

### 2.3. Calculation of Land Use Land Cover Change 

A supervised classification of land use land cover map of Nigeria with an overall accuracy of over 80% [45] was produced using a projected coordinate system and the administrative boundary of Nigeria clipped from the raster data. From this operation, 8 land use land cover classes, namely, cropland, forest, grassland, shrubland, wetland, built-up area, water body, and bare land, were classified [46]. These land use land cover classes were subjected to change detection analysis to determine the rate of change from 2000 to 2020 and the corresponding number of hectares using the spatial analyst function of ArcGIS 10.6. Subsequently, from the land use land cover for the year 2020, the cropland land cover was isolated to carry out suitability analysis. 

### 2.4. Cropland Suitability Potential Assessment

Since the focus of the study was to assess the suitability of the existing cropland in Nigeria, the following environmental factors were used for the assessment: surface soil moisture, LST, precipitation, elevation, slope, and SOC. These variables were fitted into a weighted overlay model using ArcGIS 10.6 based on a Multi-Criteria Decision Analysis (MCDA). Previous studies on land suitability for agriculture in Nigeria also used weighted values for soil and climate data calculated using MCDA and simulated in a GIS environment [23,32,47]. However, this study is a national-level assessment, and the physical variables differed by the inclusion of SOC, which is a critical component for soil fertility. While the parameters selected for this study are logical [48], the spatial dataset, such as land use land cover data, has a fine resolution of 30 m. In addition, all spatial datasets were resampled to a resolution of 30 m to increase the accuracy of the results. The steps to calculate the cropland suitability are as follows:

(i) First, the processing of the spatial datasets is explained below:
  (a) The mean surface soil moisture was downloaded from Google Earth Engine and analyzed using ArcGIS 10.6 to determine its values across the study area.
  (b) Land surface temperature (Day time LST), a product with a repeat-cycle of 8 days, was averaged and converted to degree Celsius using Google Earth Engine and analyzed using ArcGIS 10.6 to determine its values across the study area.
  (c) The spatial distribution pattern of annual precipitation was analyzed using the Inverse Distance Weighted (IDW) spatial analyst function in ArcGIS 10.6.
  (d) The digital elevation and (e) the percentage rise of slope was determined using the spatial analyst function in ArcGIS 10.6.
  (e) The spatial distribution of SOC in the country was extracted from the global soil organic carbon map, indicating SOC stock from 0 to 30 cm.
(ii) Secondly, to evaluate the assessment of the cropland suitability potential of the country, weights were generated for the six variables using Pairwise Comparison Analysis (Table 2), which is a part of multicriteria decision method, after quantifying the influence of the variables based on an individual analytical hierarchical process (AHP) as explained by Saaty (2008) [49] on a numerical scale (1–5) to indicate the suitability values over other values within the same variable (Table 3). This Analytical Hierarchy Process (AHP) is based on a hierarchical structure [50] and is effective in determining weights [44]. This implies that the higher the hierarchical value, the more suitable the potential for agriculture.
(iii) From the analyzed pairwise comparison, the weighted values of each variable were fitted into a weighted overlay model in ArcGIS using the following formula:
  LS = *n*(SSM)*_wt_*_(1–5)_ + *n*(LST)*_wt_*_(1–5)_ + *n*(Precip)*_wt_*_(1–5)_ + *n*(Elev)*_wt_*_(1–5)_ + *n*(SLP)*_wt_*_(1–5)_ + *n*(SOC)*_wt_*_(1–5)_ (1)
  where
  LS = Land suitability;
  *n* = The weighted value from the pairwise comparison;
  *wt*(1–5) = The individually weighted variables on a scale of 1–5 based on their suitability using AHP;
  SSM = Surface soil moisture;
  LST = Land surface temperature;
  Precip = Precipitation;
  Elev = Elevation;
  SLP = Slope;
  SOC = Soil organic carbon.
(iv) Lastly, to achieve the study objective, the weighted overlay model outcome was multiplied by the recent cropland land use land cover of 2020, using the spatial analyst function of ArcGIS to model the existing cropland suitability, which was reclassified into five (5) classes, namely, Very High Suitability (5), High Suitability (4), Moderate Suitability (3), Marginal Suitability (2), and Low Suitability (1). All dataset values are given in Table 3.

## 3. Results and Discussion

### 3.1. Analysis of Land Use Land Cover Changes

**Figure 2:**
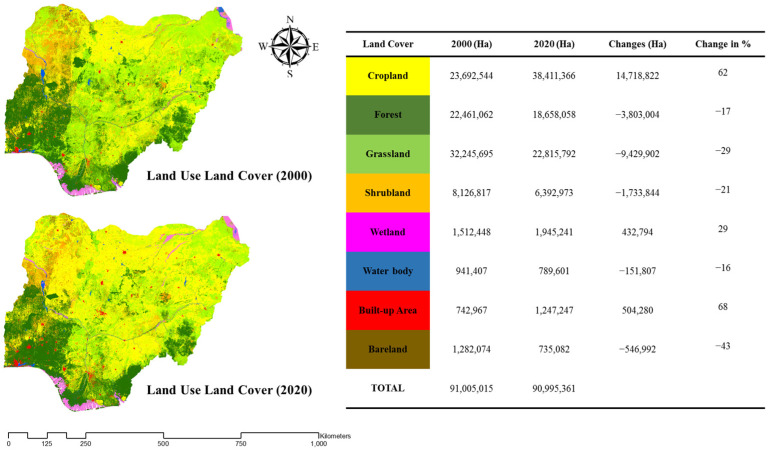
Land use land cover conversion with corresponding area in hectares from 2000 to 2020.

The land use land cover results revealed a 62% expansion rate of croplands across the country from 2000 to 2020. This change pattern indicates a significant alteration in land cover, highlighting the paramount demand for food production in Africa’s largest population. Further changes in land use land cover can be seen in the figure below (Figure 2). 

### 3.2. Environmental Suitability Potential Assessment for Agriculture

**Figure 3:**
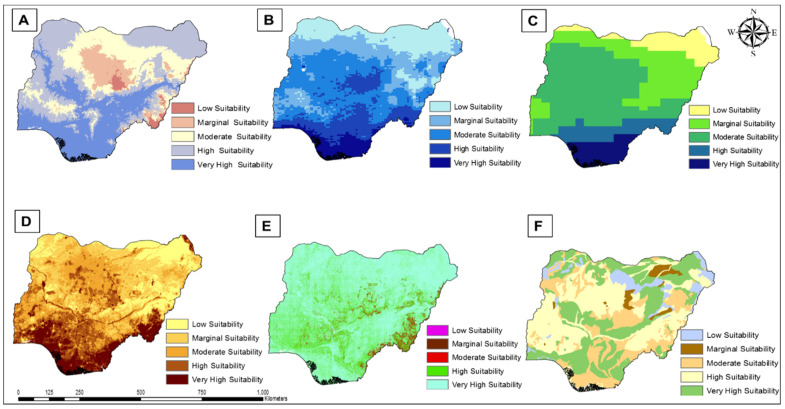
Spatial assessment of individual variable suitability across Nigeria. (**A**) Elevation, (**B**) Surface Soil Moisture, (**C**) Precipitation, (**D**) Land Surface Temperature, (**E**), Slope, and (**F**) Soil Organic Carbon.

In general, the surface soil moisture (SSM), which gives an indication of the degree of wetness or dryness of the soil and contributes to ecological functions, decreases from the South to North of the country. A breakdown shows that 7% of the land area has a very high suitability for SSM (value: 21–25) and 17% as high suitability (value: 17–20), primarily located in the South and coastal regions. Meanwhile, the North Central regions have a 33% area with moderate suitability surface soil moisture (value: 13–16). The core Northern areas have 23% and 20% of their area classified as marginal (value: 8–12) and low suitability (value: 3–8), respectively (see Figure 3). 

The spatial outcome of the LST as a proxy to areas prone to water stress and crop growth indicates that the country has land areas with 13% of very high suitability (value: 21–28 °C) and 18% within high suitability (value: 28–31 °C) LST, while 27% are under moderate suitability (value 31–33 °C). The areas with marginal (value: 33–35 °C) and low suitability (value: 35–41 °C) LST are 28% and 15%, respectively. 

The amount of precipitation decreases from South to North, and in terms of the area with sufficient rainfall, 8% of the land area falls within very high suitability (value: above 2100 mm), 10% has high suitability (value: 1600–2100 mm), 46% is moderately suitable (value: 1200–1500 mm), 26% are under marginal (value: 850–1100 mm), and 11% (value: 380–840 mm) are low-suitability, respectively. Areas with low precipitation may not encourage crop growth for water-intensive crops when solely dependent on precipitation. However, groundnuts, sorghum, and millet can be cultivated within such low rainfall areas, as they are less water-intensive crops. It is worth noting that these assessments were made without the consideration of the impact of climate change on rainfall pattern and the use of irrigation as an alternative to crop water supply for food production. This is due to low irrigation use in the country, as stated earlier. Therefore, climate variability [51] poses a risk factor for food availability, especially in Africa [52,53], where livelihood is dependent on an agricultural-based economy [54]. This is because studies have cited climate-related yield losses in Southern Africa [52] and Eastern Africa [53] (see Figure 3).

Based on elevation, 28% of the country’s land area is categorized under very high suitability (value: −40–190 m) and 38% (value: 200–360 m) as high suitability land areas for food production, implying that the topography is flat enough to carry out mechanized farming. In addition, 23% are classified as having moderate suitability elevation (value: 370–570 m), while 9% (value: 580–970 m) and 2% (value: 980–2400 m) are classified as marginal and low suitability areas, respectively, due to very high elevations. Nevertheless, some crops such as potatoes and leafy vegetables can thrive in high elevations due to the cool temperature [55] (see Figure 3). 

The areas with high slope percentage gradients that have very high suitability (value: 0–4%) and suitable areas (value: 4–24%) occupy 71% and 26.4%, respectively, while 2% are under moderate suitability (value: 25–52%), 0.6% are classified as marginal suitability (value: 53–100%), and 0.1% are highlighted as low suitability (value: above 100%) due to the steep slopes. However, the greater the percentage rise in slope, the more susceptible to soil erosion. Therefore, areas with a high slope gradient can cultivate tea and engage in livestock grazing.

The country’s terrain is mostly identified as lowlands, as most areas are moderately suitable, and it is a suitable spatial determinant for arable land [56,57]. This implies that the country has vast land areas suitable for intensive and mechanized food production. 

SOC, as an indicator of soil health, shows that 32% of the topsoil of the land area has very high suitability (value: 3.14 cm), 36% with high suitability (value: 2.02 cm), and 22% having moderate suitability (value: 1.60 cm) SOC depth. Furthermore, 3% (value: 1.15 cm) and 7% (value: 0.5 cm) account for marginal and low suitability of the land area (see Figure 3). The availability of high soil organic matter content cannot be overemphasized, as it provides nutrients and improves water availability for crops [58].

The use of organic and inorganic fertilizers has been shown to be beneficial in replenishing SOC content [59]. However, monocropping and extensive tillage, being a common practice in farming practice in Africa, negatively affect SOC stocks [58]. Nevertheless, Nigerian smallholder farmers have low utilization of inorganic fertilizers (35.4%), organic fertilizers (23.1%), and herbicides (34.7%) [60,61].

### 3.3. Cropland Suitability Assessment 

**Figure 4:**
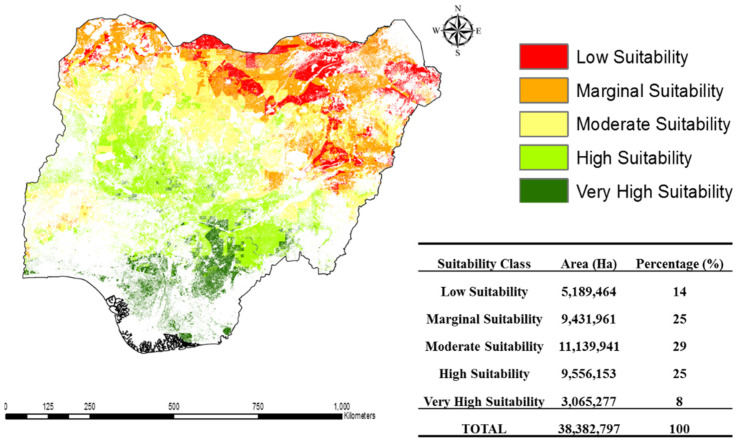
Spatial distribution of Nigeria’s cropland suitability with corresponding hectares in 2020.

From the combined influence of the selected variables, the cropland suitability was evaluated from the land use land cover of 2020 to have a more precise and current assessment of the existing agricultural potential of the country. The results indicate that 8% of the country’s existing cropland areas have very high suitability, 25% have a high suitability, and 29% and 25% are classified as moderate and marginal suitability, respectively. However, the low suitability amounts to 14% of the entire cropland. This highlights that the country has abundant arable land to be self-sufficient, considering the low percentage that is classified as low suitability (Figure 4).

To better understand the extent of existing croplands potential in Nigeria, the spatial distribution map of cropland suitability in the six geopolitical zones of Nigeria was developed. The delineation shows that very high and moderate cropland suitability levels are distributed across most parts of the South and the North Central regions of the country. Specifically, the South East has two classes of cropland categorized as very high and high suitability levels, accounting for 94% and 6% of the cropland, respectively. The South South has four classes of cropland suitability, namely, very high, high, moderate, and marginal suitable croplands of 87%, 11%, 2%, and 0.004%, respectively. The South West croplands suitability is distributed as very high (12%), high (21%), moderate (58%), marginal (9%), and low (0.04%) (Figure 4). 

A large expanse of suitable cropland is observed in the North Central with 10% being very high suitability cropland, followed by 69% with high suitability, 19% with moderate, 2% with marginal, and 0.04% with low suitability. There were more marginal suitability croplands in the North East. However, their suitability was categorized as very high suitability (1.1%), high suitability (7%), moderate suitability (24%), marginal suitability (40%), and low suitability (27%). While more moderately suitable croplands were found in the North West, the spatial distribution of their suitability is given as very high (0.008%), high (10%), moderate (44%), marginal (32%), and low suitability (14%) (Figure 4). 

The result of the cropland suitability was compared with the 2017 crop production in the six geopolitical zones of the country, considering crops such as rice, maize, cassava, yam, cowpea, onion, and tomato. The crop production data were from the Nigerian Federal Ministry of Agriculture and Rural Development (FMARD). The comparison between the cropland suitability in the six geopolitical zones and their crop production data indicate that most areas in the South East, South South, and South West have highly suitable croplands that meet the biophysical conditions for crop production, followed by the North Central regions. In contrast, most places in the North (North East and North West) have a higher share of moderate to low suitability (Table 4). As a proxy, this is reflected in the crop production of the six geopolitical zones.

The Food and Agriculture Organization examined millet and sorghum farming system and found that they are more commonly cultivated in the semi-arid regions of sub-Saharan Africa, and intercropping is prominent in the humid regions of Nigeria [62]. This represents the cropping patterns in the country and may be connected to the suitability potential of the country. The semi-arid region parts of Northern Nigeria mainly cultivate cereals such as sorghum, millet, etc., while the humid zones in the South engage in mixed cropping [63], specifically cassava, which, in Nigeria, is mainly concentrated in the South and North Central areas. The humid region of the Southern parts of Nigeria have drained and moist soils that allow for a deep penetration of root tubers, allowing them to develop efficiently [25]. In the North, there are more drier conditions, leading to timing of crop cultivation [61] (Table 4). 

Aside the cropland suitability, the choice of farmers plays a role in the cultivation of crops. For instance, a study observed that the North East and North West regions of Nigeria cultivate more maize than sorghum and millet due to the higher yield and market potential [64]. 

Nevertheless, this study infers from the spatial assessment that the South and vast areas around the North Central of the country have the propensity to support food production due to their environmental suitability. This observation is supported by their crop production in 2017, as stated in Table 4. Therefore, to maximize these potentials, the land suitability should be considered for more targeted cultivation. For instance, the expansion of rice-producing areas as land under rice cultivation is low despite the potential for expansion to increase production [65].

While the North receives minimal rainfall, especially towards the Sahel region of the country, the use of low-lying areas and flood plains called “FADAMA” for rice and vegetable cultivation is prominent [66]. However, this approach still faces suboptimal output due to low farming inputs [67]. On the contrary, a study carried out in Bansara in Ogoja Local Government Area of Cross River State, South South region of Nigeria, specifically considering land suitability for FADAMA, observed that the area had high suitability, and found that it can also support the FADAMA system of farming [68]. Since the country has a low irrigation scheme, it is recommended to adopt the use of region-specific improved varieties to boost harvest in these regions. Nevertheless, areas faced with low suitability reflect areas where land management intervention in terms of food production techniques can improve food production. 

## 4. Conclusions

To match the land suitability to the context of meeting food production demands, the combined approach of AHP and remote sensing provided insights into cropland suitability, and the following conclusions were drawn:

From spatial analysis using fine-scaled resolution data, the country has abundant land suitability potential for agriculture. Therefore, with the right support from the government, related agencies, and stakeholders, the existing cropland shows the potential of the country to target and achieve localized self-sufficiency in staple foods. Also, this approach can be replicated across regions that intend to assess their land suitability for food production.

Currently, cropland expansion is a major driving factor of land use land cover change in Nigeria, while grassland, forest, and shrublands, respectively, are being lost. Therefore, appropriate and sustainable land management systems, such as land conservation, need to be put in place to limit the impact of resource degradation arising from land cover changes. 

The crop production data support the cropland suitability results as they show that the South and North Central have a higher suitability to produce various kinds of food in abundance. Therefore, we recommend using the cropland suitability to map out food security programmes and adopt the use of region-specific improved varieties to boost harvest, considering their inherent environment potentials to alleviate the food production needs of the country.

In sum, there is the likelihood of encountering low suitability mostly within the North West and North East axes due to environmental factors. This may suggest why smallholder farmers across the country cultivate crops that can thrive in their respective localized environmental conditions. To solve this, land suitability should be considered for more targeted cultivation and as a guide for decision-making for sustainable agriculture in the country. 

Also, the outcome of this research contributes to policy consideration to strengthen and promote local smallholder farmers production capacity, while reducing the depletion of the nation’s foreign reserves from importation on food that can be locally produced to meet demand.

Lastly, the study might have been influenced by the choice of weighted variable values used for the analysis, and remotely sensed data are subject to errors that may influence the outcome of the study. Also, this study acknowledges the limitation of not considering crop-type-based assessment at the national level due to data availability as of the time of this research. Furthermore, the study assumed the non-use of irrigation as an alternative to crop water supply and a “conflict-free scenario” as constraints to land suitability, and it considered only environmental and biophysical factors that affect food production. Therefore, further studies are needed to examine, specifically, the impact of conflict and climate change on food production and crop-type-based assessment at the national level.

## Figures and Tables

**Table 1:** Summary of the data source information.

Data Source	Data	Resolution	Year
Land Use Cover Change Analysis			
National Geomatics Center of China (NGCC)	Land Use Cover	30 m	2000 and 2020
Cropland Suitability Analysis			
Food and Agricultural Organization (GSOC map)	Global Soil Organic Carbon	30 arc seconds (eq to 111.2 km)	2012
Climate Research Unit (University of East Anglia)	Precipitation	0.5° (eq to 55.66 km)	2020
Moderate Resolution Imaging Spectroradiometer (MODIS)	Land Surface Temperature	1 km	2020
Consultative Group for International Agricultural Research (CGIAR)	Digital Elevation	90 m	2000
Consultative Group for International Agricultural Research (CGIAR)	Slope	90 m	2000
National Aeronautics and Space Administration/United States Department of Agriculture (NASA-USDA)	Surface Soil Moisture	10 km	2020

**Table 2:** Weighting criteria by pairwise comparison.

Criteria	SSM	LST	Precipitation	Elevation	Slope	SOC	TOTAL	Weights	%
SSM	1	5	2	5	5	3	21	0.32	32
LST	0.2	1	3	1	0.5	0.2	6	0.09	9
Precipitation	0.5	0.3	1	5	5	1	13	0.19	20
Elevation	0.2	0.2	0.2	1	5	1	8	0.12	12
Slope	0.2	5	0.2	1	1	1	8	0.13	13
SOC	0.3	5	1	1	1	1	9	0.14	14
Total							65	1	

**Table 3:** Analytical hierarchal process of the six variables.

Variables	Value	AHP	Suitability Class
Surface Soil Moisture	21–25	5	Very High Suitability
	17–20	4	High Suitability
	13–16	3	Moderate Suitability
	8–12	2	Marginal Suitability
	3–8	1	Low Suitability
Land Surface Temperature (°C)	21–28	5	Very High Suitability
	28–31	4	High Suitability
	31–33	3	Moderate Suitability
	33–35	2	Marginal Suitability
	35–41	1	Low Suitability
Precipitation (mm)	2200–2900	5	Very High Suitability
	1.600–2100	4	High Suitability
	1200–1500	3	Moderate Suitability
	850–1100	2	Marginal Suitability
	380–840	1	Low Suitability
Elevation (m)	−40–190	5	Very High Suitability
	200–360	4	High Suitability
	370–570	3	Moderate Suitability
	580–970	2	Marginal Suitability
	980–2400	1	Low Suitability
Slope (%)	0–4	5	Very High Suitability
	4–24	4	High Suitability
	25–52	3	Moderate Suitability
	53–100	2	Marginal Suitability
	Above 100	1	Low Suitability
Soil Organic Carbon (Top Soil) (cm)	3.14	5	Very High Suitability
	2.02	4	High Suitability
	1.60	3	Moderate Suitability
	1.15	2	Marginal Suitability
	0.5	1	Low Suitability

**Table 4:** Six geopolitical zones and their local crop production (tonnes) in 2017.

	NORTH CENTRAL	NORTH EAST	NORTH WEST	SOUTH EAST	SOUTH SOUTH	SOUTH WEST
Rice	3017	1376	1993	398	464	577
Sorghum	1538	1914	3207	nil	nil	nil
Maize	3108	3145	2827	625	725	1678
Cassava	14,651	3516	4559	10,132	11,076	11,135
Yam	18,536	5445	3381	8744	10,023	7955
Cowpea	841	1146	861	307	182	539
Onion	98	551	776	nil	nil	8
Tomato	540	906	961	102	50	249
TOTAL	45,126	17,999	18,565	20,309	22,520	22,141

## Data Availability

Food Crop Production and Harvested Area Statistics 2017 https://fmard.gov.ng/; Land use land cover images (30m) http://www.globallandcover.com.
